# Supplementary figures and images for: The Effects of Endoplasmic Reticulum Stress via Intratracheal Instillation of Water-Soluble Acrylic Acid Polymer on the Lungs of Rats
Source: Int J Mol Sci. 2024 Mar 22;25(7):3573. doi: 10.3390/ijms25073573 (PMC11011863; doi:10.3390/ijms25073573)

Supplementary Figure S1

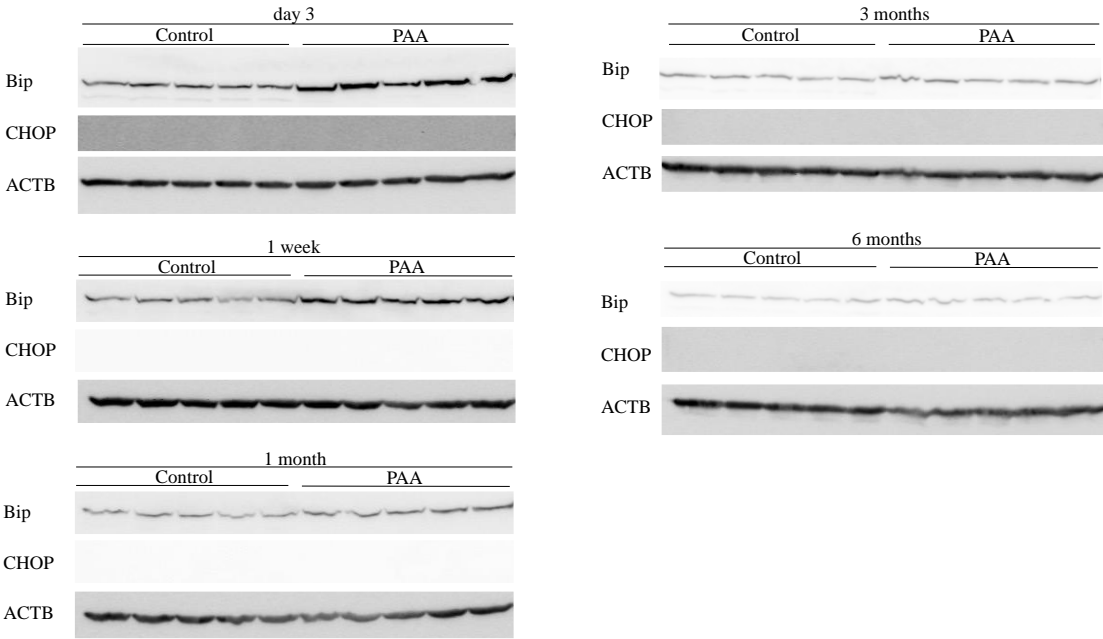

Supplement: Supplementary file 1 [file ijms-25-03573-s001.zip › ijms-2886567-supplementary.pdf]
